# New Wound Management of Driveline Infections with Cold Atmospheric Plasma

**DOI:** 10.3390/jcdd9110405

**Published:** 2022-11-21

**Authors:** Jamila Kremer, Étienne Fasolt Richard Corvin Meinert, Mina Farag, Florian Mueller, Jasmin Penelope Soethoff, Matthias Karck, Bastian Schmack, Anna Lassia Meyer, Gregor Warnecke

**Affiliations:** 1 Department of Cardiac Surgery, Heidelberg University Hospital, Im Neuenheimer Feld 420, 69120 Heidelberg, Germany; 2 Department of Cardiology, Angiology and Internal Intensive Care Medicine, Saarland University Hospital Kirrberger Straße 100, 66421 Homburg, Germany; 3 Department of Thoracic and Cardiovascular Surgery, Essen University Hospital, Hufelandstraße 55, 45147 Essen, Germany

**Keywords:** left ventricular assist device, wound infection, driveline infection, mechanical circulatory support, cold atmospheric argon plasma

## Abstract

The use of ventricular assist devices as a bridge to transplant or as destination therapy has increased. Wound complications increase morbidity in this cohort. Cold atmospheric plasma is a source of reactive oxygen and nitrogen species and can reduce the microbial load in skin wounds without negative effects on the surrounding tissue. We evaluated our cold atmospheric plasma treatment for LVAD driveline infections in a retrospective single-center study for peri- and postintervention outcome analysis. Between April 2019 and September 2019, 15 male patients were included (5 HVAD, 10 HeartMate III). The wounds were treated for a mean of 368.5 s with a reduction of bacterial load in treated wounds in 60% of patients, regardless of the pathogen. The most common pathogen was staphylococcus aureus (*n* = 8 patients). There was a significant reduction of the wound scale (scale 2.80 vs. 1.18; *p* < 0.001) plus a significant reduction in size (16.08 vs. 1.90 cm^3^; *p =* 0.047). Seven patients (46.6%) were free from any signs of local or systemic infection during 1-year follow-up. Five patients (33%) received a heart transplantation. Cold atmospheric plasma treatment is a potent, safe, and painless adjuvant technique for treating driveline infection without the need for repeating surgical interventions.

## 1. Introduction

Since the introduction of left ventricular assist devices (LVAD) for patients with terminal heart failure (HF) driveline associated infections seriously contribute to morbidity and mortality in this patient cohort [1]. In a study by Goldstein et al., up to almost 20% of patients with a LVAD developed driveline infection within one year after implantation [2]. Furthermore, infections may lead to an increased risk of stroke and adverse outcomes after stroke [3]. Inflammation and thrombosis may be promoted by bacteremia, with sepsis being associated with significantly increased odds of ischemic and hemorrhagic stroke within 15 days of septic onset [4]. In a comprehensive analysis of the MOMENTUM 3 trial focusing on infection-related outcomes, the increased number of deaths in the infection group was, in part, due to right heart failure (25%), stroke (17%), and other causes (38%). The authors state the importance of the link between infection-related complications and postoperative outcome.

Infections are not only limited to the driveline. Therefore, the International Society of Heart and Lung Transplantation (ISHLT) acknowledges the scale of LVAD-related infections. In a consensus document, the authors differentiate between VAD-specific, VAD-related, and non-VAD infection in this cohort. VAD-specific infections may be pocket and driveline infections and therefore can be localized easily, however, such infections can become systemic, with involvement of the blood stream, and lead to endocarditis [5].

In this study, we focus on driveline infections as defined by the ISHLT, which were treated by cold atmospheric pressure argon plasma (CAP). Previous studies on the antimicrobial activity of CAP showed promising effects against a wide range of pathogens in other fields [6]. Plasma medicine mainly relies on the use of CAP and its biomedical application. Cold atmospheric pressure plasma is a source of reactive oxygen (ROS) and nitrogen species (RNS) [7]. Different cellular and subcellular reactions and effects are affected by ROS and RNS. Since the sensitivity to these alterations by ROS and RNS is different for several cell types, there is a selective use of CAP in the clinical field [8]. In a study of radial forearm free flap with exposed flexor tendons, CAP treatment achieved complete wound repair in terms of the absence of tendon exposure within a mean treatment time of 1.1 weeks, with neither undesirable side effects being observed nor any signs of inflammation or infection [9].A German study group treated 36 patients with chronic infected skin wounds using CAP at the department of Dermatology of the Hospital Munich Schwabing. They showed a reduction of one third of the bacterial load in treated wounds, regardless of the type of bacteria [10].

Herein, we present our detailed data of driveline infection treatment with CAP, demonstrating of safety and efficiency of CAP in this field.

## 2. Materials and Methods

In a retrospective single-center study, we evaluated our LVAD patients who received CAP for the treatment of driveline infections for peri- and postintervention outcome analysis. Between April 2019 and September 2019 we treated 15 male patients with driveline infection with CAP at our institution. Of these, five patients had a HVAD (Medtronic, Dublin, Ireland) and 10 patients had a HeartMate3LVAD (Abbott, Chicago, IL, USA) driveline infection. A detailed description is listed in Table 1.

The patients were treated with CAP either in the outpatient VAD clinic or during an inpatient hospital stay. Bacterial species were detected using standard bacterial swabs on a regular basis. Concomitant antibiotic treatment was administered in accordance with the antibiogram of the pathogens found in microbial analysis from wound swabs of each patient. Wound dressings were performed under sterile conditions, as mentioned below. We used an Adtec MicroPlaSter (Adtec Healthcare, Hounslow, Middlesex, UK) for CAP administration. The device was loaned to our department free of charge for a limited period of time in 2019. This study complies with the Declaration of Helsinki and was approved by the ethical committee of the University of Heidelberg, S-144/2020.

### 2.1. Cold Atmospheric Argon Plasma Treatment

The CAP device was positioned directly above the debrided driveline infection site, as close as possible to the wound. Depending on the wound depth and the level of wound healing, treatment duration varied between 120 and 480 s, with longer treatment times needed for deeper wound infections. Time allows the cold plasma gas to travel down the gaps through the different tissue layers towards the biofilm site. The CAP device can cover a treatment area of up to 12 cm^2^ per application. In general, one application per session was sufficient. The physical mode of action delivered during CAP treatment ensured that all bacteria were targeted and destroyed, regardless of their resistance profile or presence of biofilm.

#### 2.1.1. Wound Dressing

Wound dressing changes were performed under sterile conditions. The driveline exit sites were either cleaned with Octenisept^®^ (Schülke & Mayr GmbH, Norderstedt Germany) or Granudacyn^®^ (Mȯlnlycke Health Care GmbH, Düsseldorf, Germany) and on two occasions, with Prontosan^®^ (B. Braun Melsungen AG, Melsungen, Germany). Wound dressing was performed with Aquacel Ag + Extra (ConvaTec GmbH, München, Germany) and Mepilex (Mȯlnlycke Health Care GmbH, Düsseldorf, Germany). Three patients were treated with Cutimed^®^ Sorbact^®^ (BSN medical GmbH, Hamburg, Germany) instead of AquacelTM Ag + ExtraTM.

To calculate the average cost of driveline infections, we analyzed our most recent economic data from 2020 and evaluated the differences between dressing changes of vacuum-assisted therapy in the operating room or in the ambulatory setting versus CAP treatments.

#### 2.1.2. Statistics

All statistical analyses were performed using the IBM SPSS Statistics version 25 software (SPSS, Chicago, IL, USA). Normally distributed continuous variables were reported as mean ± standard deviation and were compared using a two-tailed t-test. Categorical variables were reported as frequencies and percentages and were analyzed using the x^2^ test. Survival was calculated using the Kaplan–Meier method. The threshold for significance was set at *p* < 0.05.

## 3. Results

All 15 patients were initially admitted to our normal ward because of wound infection on the driveline exit site. After initial surgical debridement and/or vacuum-assisted wound closure (VAC), further treatment was continued with CAP treatment alone.

Altogether, 120 CAP treatment sessions were performed in our 15-patient collective. The average number of CAP sessions was 7.7 (1–17 sessions/patient). The wounds were treated for a mean of 368.5 (120–480) seconds of CAP in each session. The median interval between LVAD implantation and first CAP session was 367 days (33–2011 days). Median duration of CAP treatment was 42 days (1–117 days). A detailed overview of the CAP sessions is presented in Table 2.

We found a reduction of bacterial load in CAP-treated wounds in nine (60.0%) patients, regardless of the pathogen. The most common pathogen was staphylococcus aureus (*n* = 8 patients, 53.3%). Two patients showed driveline infection with staphylococcus epidermidis additive to the staphylococcus aureus and two patients had an enterococcus faecalis infection. Other bacteria found in the wound swabs included: *corynebacterium species, candida parapsilosis, proteus mirabilis, serratia mirabilis, pseudomonas aeruginosa,* and *citrobacter species.*

**Figure 1:**
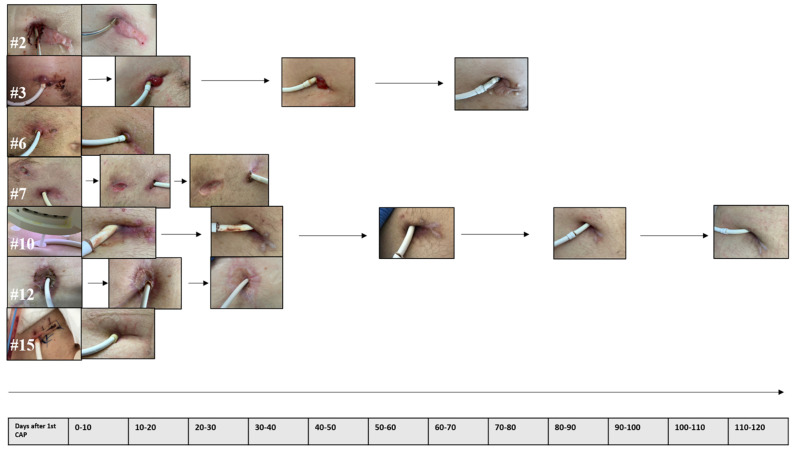
Wound history in seven patients with complete wound healing after CAP treatment alone.

There was a reduction in the wound scale according to our institution’s wound classification, Table 3, comparing before and after CAP treatment (scale 2.80 vs. 1.18; *p* < 0.001), as well as a significant reduction in wound size (16.08 vs. 1.90 cm^3^; *p* = 0.047), Figure 1.

### 3.1. Mortality, Survival, and Follow-Up

No adverse effects occurred and treatment was well tolerated.

Five patients (33%) received a heart transplantation after a median of 116 days (2–553 days) after the last CAP treatment session, with no further complications around the former driveline exit site during follow-up. All five patients achieved a clinically stable condition with no additional prolonged antibiotic treatment despite driveline infection pre-transplantation. One of these patients had developed another driveline infection prior to transplantation but was then successfully transplanted, resulting in complete healing of the former driveline exit site.

Three patients (20%) developed recurring driveline infection during follow-up after completed CAP treatment, two patients again underwent treatment with a vacuum-assisted wound dressing. Both patients were later discharged and are seen in our outpatient clinic for regular follow-up. One of the patients is currently on our transplantation waiting list, and one has redacted his consent for a heart transplant, for now. The third patient with a recurrent wound infection is being treated with adequate wound dressing changes and calculated antibiotics; however, this patient is not transplantable due to underlying contraindications.

The remaining seven patients (46.6%) were free from any signs of local or systemic infection during 1-year follow-up.

Survival rate during follow-up was 100%, with a cumulative follow-up time of 6162 days.

### 3.2. Calculated Costs

In 2020, we performed 259 VAC dressing changes at our center; 192 were performed in the operation room (OR). The average cost to rent a VAC pump at our hospital is 40 euros per pump per patient. A sterile VAC dressing change in the OR costs a total of 320 euros, including OR time and staff fees, plus wound dressing material and personal protection equipment for nurses and physicians, excluding available infrastructure. The calculated cost of one Adtec MicroPlaSter CAP application, including the investment in the device and annual maintenance, as well as costs of nurses and physicians, plus wound dressing material and personal protection equipment, is currently calculated at 163 euros per application per patient.

## 4. Discussion

In recent years, implantation of ventricular assist devices has increased markedly in number, either as a bridge to transplant or as destination therapy. Complications became apparent and presented a new challenge in the aftercare of this special patient cohort [11]. Bleeding and thrombosis remain the major complications, however device infection is reported in up to 60% of cases. Infection is listed as the fourth most common cause of death within one year after implantation (228 of 9.781, or 8.8%), following neurologic complications (15.6%), multi-system organ failure (15.6%) and discontinuation of LVAD support (10.4%) [12]. Driveline infections are the most prevalent infections [13]. Patients with infections and wound healing disorders are often hospital-bound and morbidity and mortality are elevated. The risk of severe bloodstream infection is hign since foreign material colonization and multiresistant pathogenes are amnipresent. Deep driveline infection is best assessed with 18F-FDG PET/CT scans or 67 gallium or 111 In-leukocyte SPECT/CT [14,15,16]. VAD-specific infections may be pocket and driveline infections and therefore can be easily localized; however, such infections can become systemic, with involvement of the blood stream, and lead to endocarditis and mediastinitis [5,17]. Once there is manifestation of driveline infection, surgical management of the driveline differs depending on whether the infection is superficial with minimal skin irritation, or deep, involving infection of the surrounding tissue. There are sparse recommendations regarding how to treat these wound infections. Infection-resistant drivelines with antibiotic impregnation were manufactured. Furthermore, improving the fixation technique to reduce trauma on the driveline exit site by improving local vascular supply or by using muscle flaps once there is severe tissue damage are recommended [18,19].

Vacuum-assisted therapy is a treatment method that uses sub-atmospheric pressure (50–125 mmHg vacuum) to treat different kinds of wounds. With a sterile polyurethane foam sponge placed into the wound cavity, the wound is later covered by a thin adhesive film and is sealed from the outside. This seal is critical for creating the vacuum for the wound. It can improve tissue granulation and local capillary circulation [20].

However, surgical vacuum-assisted treatment is time-consuming and demands a lot of resources. Often these dressing changes have to be performed in the operating room to guarantee sterile wound cleaning and adequate pain relief therapy. The estimated average cost for each surgical site infection in the United States is about $2.734, according to a study by Jarvis W. et al. In the article the authors state that diagnosis-related group systems cost hospitals from $583 to $4886 for each nosocomial infection [21]. In the United Kingdom, the NHS calculated that some £3.2 billion were spent on the treatment of 40% of wounds with delayed healing [22,23]. The material costs of diagnostic equipment and devices for wound care as well as special wound dressings are often taken into consideration; however, the costs of the workforce, the treating physicians’ and health care professionals’ time, duration of care expenditure, and the occurrence of complications are generally neglected [24]. A prolonged hospital stay, and a chronic wound history take their toll on the patients’ physical and mental states as well as their quality of life. The reduction of pain and inconvenience, the reduction of exudate, and the appearance of the wound are objective criteria, from the patient’s point of view, which might influence the patient’s compliance and comfort [25].

In our calculation, we showed that an investment in the Adtec MicroPlaSter CAP would reduce our driveline infection costs by 50%. Furthermore, we found a reduction of bacterial load in CAP-treated wounds in most of our patients, regardless of the pathogen. In the treatment of chronic wounds, CAP is gaining application to a wider field. It has been shown that CAP can reduce microbial load without negative effects on the surrounding tissue [6]. The effects were first discovered in the 1990s [26]. Cold atmospheric plasma reduces bacteria viability predominantly by formation of UV radiation and induction of ROS and RNS [8,27,28,29]. For this clinical evaluation, we focused on the effect of CAP on microbial pathogens and the role of redox species on wound healing in patients with driveline infection. It was demonstrated that short treatment times had a cell-stimulating effect, whereas higher treatment durations could lead to cell apoptosis and inactivation of cellular mechanisms [30]. Persson et al. further demonstrated that no microbial resistance to CAP treatment was found, nor were there any upregulated immune reactions. Instead, they described that the short, tissue-tolerable CAP treatments disarmed the important virulence factor, supporting the usefulness of CAP application for combatting skin- or surgery-related bacterial infections [31].

Pathogens causing early and late infections in LVAD patients are predominantly bacteria, while fungi are less prevalent. The most prominent bacterial pathogens are staphylococcus aureus and epidermidis, with an infection rate of almost 50% of all LVAD infections. These species colonize the skin and therefore adhere to the driveline and the newly implanted device and form a biofilm. The second-most infectious agents are enterococcus species. Pseudomonas and other Gram-negative species are responsible for around 30% of VAD infections, and fungal infections are only noted in up to 10% [11,32,33,34,35,36,37]. The first use of CAP in driveline infection was described by Hilker et al. in a case report. The authors applied CAP through the kinPen^®^ MED plasma jet device (neoplas tools GmbH, Greifswald, Germany). Their patient was a 66-year-old HVAD patient who received 1 min of CAP exposure once a day for 12 consecutive days. After an additional treatment period of 4 weeks in the outpatient clinic, the local infection was completely healed without any signs of exudation or recurrence of the infection [38].

Herein, we present our results with CAP treatment in the surgical therapy of acute driveline infections in LVAD patients. Through repeated CAP sessions we demonstrated a reduction in wound size and bacterial growth. Five out of 15 patients with driveline infection were successfully transplanted without any further infections on the former exit site. Even more importantly, 7 patients who had not undergone heart transplantation thus far, showed complete healing of their driveline infections without recurrence. This is a rare success in wound infections in the presence of foreign material and non-biologic surfaces. During follow-up we did not see any adverse effects and CAP treatment was well-tolerated. It enabled most patients to stay out of the hospital in ambulatory care.

While our study only reports on 15 cases, limiting the power of our observations, we found decreased bacterial growth in semi-quantitative assay analysis but could not demonstrate statistical significance due to a lack of numerical data. Nevertheless, this fact is coherent with data from Isbary et al. published in 2010 [10]. The authors were able to demonstrate that the effect of antimicrobial activity against bacteria was present regardless of the species of the bacteria or the antibiotic resistance level.

Our results are nevertheless compelling, since wound treatment with CAP in patients with driveline infection was universally applicable painless, and less time-consuming than conventional surgical wound treatment with, for example, vacuum-assisted therapy.

## 5. Conclusions

Cold atmospheric argon plasma treatment is a potent, safe, and painless additional technique for treating driveline infection without the need for repeated surgical interventions during the initial course of treatment. This study showed effectiveness and feasibility for patients with driveline infection and thereby serves as a basis for further studies with a larger number of patients and for studies directly comparing the CAP method with conventional surgical therapy in a randomized investigation.

## Figures and Tables

**Table 1:** Patient demographics.

Characteristics	
Number of patients (*n*)	15
Age (y)	50.5 ± 13.2
Gender (male)	15
Devices:	
HeartWare HVAD	5 (33.3%)
HeartMate III	10 (66.7%)
Height (cm)	176.3 ± 6.5
Weight (kg)	83.4 ± 18.5
BMI	26.2 ± 5.4
Diabetes mellitus	5 (33.3%)
Arterial hypertension	9 (60.0%)
Smoking	12 (80.0%)
Dyslipoproteinamia	6 (40.0%)
Renal insufficiency	6 (40.0%)
COPD	2 (13.3%)
Peripheral artery disease	1 (6.7%)
Time from LVAD implantation to first CAP (d)	586 ± 550
Number of CAP sessions (*n*)	7.7 ± 5.6
Duration of CAP treatment (d)	46.5 ± 37.2
Complete healing (*n*)	7 (46.7%)

Continuous data are presented as means with standard deviation, and categoric data as number (%). BMI, body mass index; COPD, chronic obstructive pulmonary disease; CAP, cold atmospheric argon plasma.

**Table 2:** Overview CAP treatment sessions.

	Device	Sessions (*n*)	Treatment (d)	Duration (min)	Species	Wound Result
#1	HVAD	2	3	240–480	*S. aureus*, *Corynebacterium species*	Successful HTX
#2	HVAD	8	20	240–480	*S. aureus*, *S.epidermidis*, *S. lugdunensis*, *Corynebacterium accolens*	Complete healing
#3	HMIII	11	81	240–480	*S. aureus*	Complete healing
#4	HMIII	11	54	240–480	*Candida parapsilois*	Successful HTX
#5	HMIII	8	24	240–480	*S. aureus*	Wound reduction with new VAC-treatment during follow-up
#6	HMIII	1	0	480	*S. aureus*	Complete healing
#7	HMIII	4	21	240	*S. aureus*	Complete healing
#8	HMIII	2	53	480	*Pseudomonas aeruginosa*	Wound reduction with new VAC-treatment during follow-up
#9	HMIII	17	66	480	*S. aureus*, *E. faecalis*, *Candida parapsilois*, *Corynebacterium stratium*, *Proteus mirabilis*, *Pseudomonas aeruginosa*, *Serratia mirabilis*	Successful HTX
#10	HMIII	15	117	120–480	*S. haemolyticus*	Complete healing
#11	HVAD	14	109	240–480	no identified pathogen	Successful HTX
#12	HMIII	14	37	240–480	*S. epidermidis*, *Citrobacter koseri*	Complete healing
#13	HVAD	3	42	120–240	*E. faecalis*, *Proteus mirabilis*, *Citrobacter freundii*	Complete healing with reoccurrence of DLI after 458 days
#14	HVAD	5	71	240	*S. aureus*	Successful HTX
#15	HMIII	1	0	120	no identified pathogen	Complete healing

DLI, driveline infection; E, Enterococcus; HMIII, HeartMate III; HVAD, Heartware HVAD; S, Staphylococcus.

**Table 3:** Heidelberg driveline wound classification.

Grade	Wound Description
0	Adequat wound healing with no signs of skin irriation. No fluid drainage. No tenderness.
1	Sufficient wound healing. Slight skin irration with redness. Beginning of tenderness and slight wound drainage.
2	Early skin retraction. Wound drainage. Extension of hyperthermic redness around the driveline.
3	Extention of skin retraction. Hypertrophic granulation/granuloma pyogenicum. Severe wound drainage. Skin tenderness and pain.
4	Extensive wound granulation with subsequent skin arosion. Open wounds around the cannulas. Extreme tendernss and pain.

## Data Availability

The data presented in this study are available on request from the corresponding author.
